# A Dive Into Cerebellar Dysfunction, Motor Deficits and GABAergic Signaling in Down Syndrome

**DOI:** 10.7759/cureus.79623

**Published:** 2025-02-25

**Authors:** Shannon Feely, Misleydi Rios Rodriguez, Alyssa Shannon, Serena Young, Justin Peter Rosales, Gurjinder Kaur

**Affiliations:** 1 Department of Basic Biomedical Sciences, Touro College of Osteopathic Medicine, Middletown, USA

**Keywords:** cerebellum, gaba, granular cells, hyperpolarization, long-term potentiation, motor coordination, motor deficits

## Abstract

Down syndrome (DS) mouse models and DS human fetus studies clearly indicate severe neurogenesis impairment in the cerebellum. Clinical features of DS include motor dysfunction and cerebellar hypotrophy, with a particularly marked decrease in the number of granule cells (GCs). GCs are crucial for managing sensory communication within the cerebellum via mossy fibers and their interactions with Purkinje cells (PCs) and inhibitory interneurons. The current review discusses cerebellar alterations in DS and its impact on GABAergic transmission, bringing to light the impact on GABAergic signaling and its role in motor coordination dysfunction observed in individuals with DS. The findings highlight the significance of GABAergic transmission in the pathophysiology of DS and its potential as a target for therapeutic innervation. Moreover, understanding the disruptions in GABAergic signaling may provide insights into developing novel treatment strategies.

## Introduction and background

Down syndrome (DS) is an aneuploidy that affects 1 in 700-1100 births per year globally [1-4]. DS undoubtedly results in neurological and other organ system modifications. While clinical features of DS vary, cognitive impairments remain a hallmark of this disease, contributing to a majority of intellectual disabilities [3,5-8]. Trisomy 21 affects the patient’s cognitive function, particularly in learning, memory, and communication skills [6,9]. However, there are additional ways DS patients are affected, such as developing young-age Alzheimer's disease (AD), congenital heart defects (e.g., atrioventricular septal defects, ventricular septal defects), seizures, epilepsy, and strokes. Other less extensively studied DS conditions are associated with the musculoskeletal system, motor, and coordination problems such as gait abnormalities [10]. Compromised motor coordination in DS patients is apparent as insufficient fine motor control and hindrance of acquisition of gross and fine motor skills [11].

In patients with DS, neurodevelopmental defects are evident from birth as volume reduction in specific brain regions, a decline in brain weight, and an altered number of neurons, dendrites, and dendritic branching have been noticed [1,12-14]. DS patients appear to have altered developmental trajectories of their cerebellum [15]. Individuals with DS have a small and hypocellular brain and suffer from a disproportionate reduction in the number of granule cells (GC) in their cerebellum [7,16-20]. Similarly, in the DS transgenic mouse model Ts65Dn, cerebellar volume, Purkinje cell (PC) linear density, and GC density were found to be significantly decreased [7,17,19-24]. The reduction in cerebellar volume, PC linear, and GC density in the Ts65Dn mouse model has significant functional implications. These structural abnormalities likely contribute to motor impairments, such as hypotonia, delayed motor milestones, and gait abnormalities, observed in DS individuals. Additionally, due to the cerebellum's role in cognition, these deficits may also be linked to impairments in learning, executive function, and adaptive behaviour observed in DS patients.

Furthermore, changes in several neurotransmitters and their cognate receptors have been demonstrated in DS individuals [14,25-30]. Specifically, decreased concentrations of glutamate, aspartate, serotonin, norepinephrine, and dopamine had been found in postmortem tissue samples from several brain regions of adult DS patients [25,28,31,32]. Additionally, studies have found decreased levels of γ-aminobutyric acid (GABA) in post-mortem tissue samples from various brain regions of adult DS patients [2,25,28,31-33]. In postmortem studies, quantification among GABA receptors in DS patients versus controls shows a significant reduction of GABA receptors found within the hippocampus and an imbalance in the GABA receptors present in patients with DS [5,13]. In addition, multiple studies have shown that cognitive impairment is associated with excessive levels of neuronal inhibition [2,5,34,35].

Motor deficits are among the most frequently occurring features of DS. Individuals with DS exhibit disturbances in the dynamics of movement production and postural control that are thought to have a significant impact in delaying their acquisition of motor skills. The origin of these deficits has been hypothesized to be cerebellar in nature [36]. Hypotonia and dynamic motor dysfunction are practically universal in DS. Equilibrium and motor coordination impairments have been very well-documented in Ts65Dn mice [36]. Earlier studies have also shown that cerebellar GC exhibits a tonic GABA current mediated by extrasynaptic GABAA receptors [37]. Autoantibodies against GABA-related molecules have been found in some cerebellar ataxic patients, and the GABA-mimetic drug seems to be effective in improving motor coordination in some ataxic patients [38]. Although these alterations in brain structures are evident, the mechanism of synaptic dysfunction leading to DS patients’ impairment in motor coordination impairments is still unclear. The vast majority of research on DS mouse models has been focused on the cognitive and developmental qualities that define the trisomy, however, research reports targeting cerebellar dysfunction and motor coordination deficits are meagre.

This review aims to shed light on the cerebellar deficits in DS models and explore the connection between these abnormalities and dysfunction within the GABAergic pathway in the hypocellular cerebellum. To identify relevant studies, we conducted a literature search using databases accessible through our institution, primarily Google Scholar. The search strategy included key terms such as Down syndrome, ataxia, GABAergic signalling, and cerebellar dysfunction. Articles were selected based on relevance to DS-related motor coordination deficits and GABAergic pathway involvement.

## Review

Neuroanatomic structural abnormalities

New developments in DS research include fetal and infantile magnetic resonance imaging (MRI) to examine fetal brain development, infantile neuroanatomy, and diffusion tensor MRI (dtMRI) to investigate cortical connectivity in patients [1,23,39,40-44]. With these new advances, we have learned that even considering the short stature and reduced birth weight in DS neonates, their brains are 20% smaller with reduced volumes of the frontal and temporal lobes, cerebellum, and hippocampus [1,17,18,41,45]. Recent advances in imaging such as diffusion tensor MRI (dtMRI) have allowed for a more granular exploration of white matter connectivity in Down syndrome. Studies have demonstrated decreased cortical connectivity in frontal-temporal circuits, which may exacerbate DS patients' cognitive and motor impairments [39,44]. Additionally, in vivo imaging in fetuses and neonates provides critical insights into early-stage developmental disruptions [1,42]. This difference in brain volumes appears after 28 weeks gestation [40,41]. Patients with DS exhibit less complex cortical folding with more smooth gyri, reduced cortical surface area, and increased cortical thickness. This difference in cortical folding becomes apparent at 31 weeks gestation [1]. Histologically, neurons with abnormal neuronal distribution are reduced throughout the cortex, especially in cortical layers II and IV [17].

Reduced gray matter volume is observed on dtMRI in the medial frontal regions, inferior olives, and the cerebellum in DS patients aged 6-24 as compared to age-matched controls [39,44]. Children with DS show increased dendritic branching and dendrite length than their counterparts up to six months but then show decreased branching for the rest of their lives [17]. There is increased apoptotic cell death and a 20-50% reduction in neurons from birth to five years of age in DS patients [1,17]. This phenomenon is well-observed in post-mortem studies of second-trimester fetuses, which show reduced hyperplasia alongside apoptosis, leading to fewer neurons in the neocortex, hippocampus, and cerebellum [1,17].

Histologically, from 17 weeks onward in DS fetuses, a reduction in the number of dividing cells in the dentate gyrus and periventricular white matter is observed [17,46]. In a dtMRI study of children with DS aged two to four years old, decreased myelination was found in the supratentorial white matter tracts, which corroborates current theories of delayed myelination in early childhood and defective oligodendrocyte differentiation and myelination in children with DS [1,47]. A decreased population of nodes of Ranvier was demonstrated at postnatal day 30 in Ts65Dn mice, showing a decrease in white matter tracts of the spinal cord as well; however, no significant difference in nodes of Ranvier was observed at postnatal day 60 or 10-11 months [48]. MRI studies have shown that children and adolescents with DS will also reduce white matter volumes overall [18,44,49]. Thus, these white matter tract changes may cause a developmental delay but may not persist into adulthood [48].

Individuals with DS have a small and hypocellular brain and suffer from a disproportionate reduction in the number of GC in their cerebellum [7,16-20]. Signals to the cerebellum are transmitted via mossy fibers through GCs, which send those signals to PCs and inhibitory neurons; variability in the number of GCs, synaptic transmission, or intrinsic electrical characteristics are plausible to alter cerebellar processing [18,37,50]. Similarly, in the DS transgenic mouse model Ts65Dn, cerebellar volume, PC linear density, and GC density were significantly decreased [7,17,19,20,22-24]. In particular, noted hypocellularity of the granular and periventricular zones may impair cerebellum function [17,51]. GCs typically represent 95% of all neurons in the adult cerebellum [51]. In a study of Ts65Dn mice, PCs were reduced in density by 10% compared to their euploid counterparts [52]. In a study of Ts65Dn mice, GCs were reduced in density by 80% compared to their euploid counterparts [2,19]. This reduction in GCs is the lead reason for the smaller size of the cerebellum in DS patients [11]. This is the most parallel comparison to DS patients, as the Ts65Dn model is the most similar model of note [23]. With fewer direct genes involved, the Ts1Cje mice had similar decreased cerebellar volume and GC density to the Ts65Dn mice; however, they did not have reduced PC densities [23]. The decreased size of the cerebellum was significant in these models, with an 88.8% decrease in the Ts1Cje model and an 88.1% decrease in the Ts65Dn model [23].

Despite abundant cognitive dysfunction research in areas such as hippocampal learning, plasticity, and neurotransmitter systems, investigation of cerebellar anomalies and their relation to motor coordination remains limited. Numerous mouse models, such as Ts65Dn, Ts1Cje, and Ts2, have successfully demonstrated cognitive anomalies with great effort and specificity. Most notably, the Ts65Dn mouse model proved to be an exceptional tool used by researchers to gain insight into the mechanisms responsible for these cerebellar anomalies [5,15,22,53-55]. Ts65Dn mice display several anomalies analogous to DS patients, including cerebellar dysfunction, motor dysfunction, and a small cerebellum [8,20,35]. The Ts1Cje mouse model carries a segmental duplication of chromosome 16, which is analogous to the syntenic region orthologous to human chromosome 21, resulting in phenotypic development similar to DS. Ts1Cje mouse models exhibit reduced cerebellar volume, leading to cerebellar and motor dysfunction [3,23,56,57]. The Ts2 model is like the Ts1Cje model, where a different part of chromosome 16 is duplicated, analogous to chromosome 21, and exhibits more severe cerebellar and motor dysfunction [58]. This review aims to shed light on the cerebellar deficits in DS models and explore the connection between these abnormalities and dysfunction within the GABAergic pathway in the hypocellular cerebellum. Earlier, we discussed various aspects of DS patients; now, we will hone in on cerebellar involvement and GABA connections.

Postnatal maturation of cerebellar GCs in rodents involves increased expression of specific GABAA-R subunits and the development of a tonic current generated by the repeated opening of extrasynaptic GABAA-R channels [37,50]. A study looking at tonically active GABAA receptors in Ts65Dn showed that the control of the electrical properties of cerebellar GCs by tonically active GABAA receptors was debilitated compared to wild-type mice [19,37,50].

Motor coordination disruption in Down syndrome

Motor development is delayed in children with DS, most notably standing position and walking ability [59-63]. Additionally, it has been suggested that abnormalities in brain sizes, such as reduced corpus callosum and cerebellum size, lead to psychomotor dysfunction in DS patients. Adaptive strategies, such as treadmill training and core strengthening exercises, have been shown to improve motor outcomes in individuals with DS significantly. Ligament laxity, hypotonia, and cognitive delay caused by DS directly influence the development of compensatory strategies in movement and postural control [59-61]. Moreover, interventions utilizing virtual reality technology demonstrated improvements in motor control and postural stability, with effects more pronounced when initiated during early developmental periods [62,64]. These findings advocate for personalized therapeutic regimens targeting the unique biomechanical and neurological deficits in DS. For example, smaller frontal lobe volumes cause issues with cognitive deficits, gait quality, and voluntary activities, mainly in adulthood [59]. The center of gravity is more anteriorly positioned, resulting in an anticipated ankle plantar flexion, likely due to the reduction in force and to a flat feet condition.

Furthermore, DS patients have more significant hip and ankle stiffness than healthy controls. Children with DS also displayed increased angular impulse and stiffness when walking on the treadmill. This stiffness causes an increase in hip flexion throughout the gait cycle [65]. During childhood, there is a more considerable variability in gait parameters. However, after age 12, those with DS reduce their range of motion to compensate for muscle weakness and allow for better postural control [65]. This reduction in gait velocity and step length and increased step width provide stability. Individuals with DS show more significant body sway in the mediolateral direction, which results in an increase in step width as a way to maintain the center of gravity. These adaptive strategies can be corrected in gait maturation in patients with DS if proper intervention is provided early [65]. Balance and motor functions are interrelated; therefore, both should be evaluated together in physical therapy sessions [59].

Additionally, core and treadmill training and adapted dance programs were beneficial for motor control in DS patients [63,64]. More creative interventions, such as virtual reality, can positively affect postural control, improving motor coordination, most notably postural control with eyes closed [62]. As technology advances further, more data on treatment options will become available to combat motor impairment in children with DS.

GABAergic hypothesis

GABA is the human brain's primary inhibitory neurotransmitter receptor [66]. These receptors are assembled from a combination of protein subunits in a pentameric complex, of which thousands of combinations are possible [67]. A proposed mechanism implicated in DS is the GABAergic neuron's transition from normal depolarization to a hyperpolarization mode during the second postnatal week of development within the hippocampus, cortex, and cerebellum [33,68]. Emerging evidence suggests that altered GABAergic neurotransmission contributes to DS's disrupted cerebellar architecture and functionality. Overexpression of GABA alpha-5 receptors has been implicated in reduced long-term potentiation (LTP) and motor coordination deficits in Ts65Dn mouse models [5,34]. Pharmacological interventions that modulate GABAergic activity, mainly through receptor antagonists, have shown promise in restoring synaptic plasticity and ameliorating motor dysfunctions. This developmental switch of GABA action from depolarization to a hyperpolarization mode can be observed in semi-intact hippocampal preparations and cellular culture [33]. This event is marked by GABA transitioning from an active depolarization state to a hyperpolarized state modulated by the outward flow of chloride ions [33,68]. This developmental milestone is markedly delayed in conditions such as DS, as exemplified using Ts65Dn mice [33]. In the adult Ts65Dn mice, a genetic modification of the GABAergic neuron predisposes long-term potentiation (LTP) reduction as inhibition is favored [15,33,69]. Inhibiting the effect of GABA at GABA alpha-5 receptors (5GABAARs) can reverse electrophysiological, morphological, and cognitive deficits of the Ts65Dn mouse model [49]. Earlier studies have demonstrated that hippocampal LTP is reduced in Ts65Dn mice in a GABAA receptor-dependent manner, and selective GABA and NMDA receptor antagonists can reverse functional and neuro-morphological deficits of Ts65Dn mice by promoting brain plasticity [53]. Moreover, it was demonstrated that the blockade of GABAA receptors rescued hippocampal LTP and changed learning deficits in Ts65Dn mice, reinforcing the potential therapeutic use of these particular antagonists to treat cognitive dysfunction in DS [70].

GABA receptors

Among the GABA receptors, GABAAR has been the most substantially researched. The vitality of GABAAR lies within its presence within the ligand-gated chloride channels [71]. Recent clinical trials show that activation of GABAAR leads to hyperpolarization of the membrane potential of neurons from the influx of negative ions and shunting due to the increase of membrane conductance [15]. Previous studies have focused on the 5GABAAR and its involvement in the hippocampus, relating to learning and memory. The alpha 5 subunit engrosses up to 25% of the receptors within the hippocampus [72]. Vital to learning and memory, while also playing a role in synaptic phasic inhibition, the alpha 5 subunit exemplifies the magnitude GABA receptors withhold in understanding cognitive disorders, dysfunctions, and pharmacological modalities [72,73]. The 5GABAAR is most concentrated in the hippocampus, where it mediates a tonic chloride current and contributes to GABAergic inhibitory synaptic currents [72,74]. The alpha 5 subunit effect on hippocampal function further extends into cognitive disorders, including DS [72].

Additionally, dendritic inhibition has been noted to be mediated by 5GABAAR, leading to deficits in synaptic plasticity [69]. Impaired GABA inhibition within the hippocampus was combated by decreasing expression of 5GABAAR and led to a decrease in cognitive dysfunction [4,75]. Although there is limited research on the alpha 6 subunit (6GABAAR), nearly 45% of GABA receptors within the cerebellum were found to contain the alpha 6 subunit [2,19,76]. These receptors are located at both synaptic and extrasynaptic sites, mediating both phasic and tonic inhibition of GC, respectively [19]. Overstimulation of the extrasynaptic 6GABAARs leads to motor incoordination, ataxia, dystonia, and epilepsy while a loss of stimulation is noted to cause cognitive dysfunction [19].

GABAergic alterations in the cerebellum

GABAergic over-inhibition has been identified as a contributing factor to the cognitive deficits observed in individuals with DS, with studies suggesting alterations in the cerebellum as a critical region of impact [16]. The cerebellar transcriptome in DS models demonstrates global disruption, highlighting the significant role of GABAergic neurotransmission in cerebellar development and function [52]. Additionally, research on the Ts65Dn mouse model has revealed that the developmental switch of GABA polarity is delayed in the cerebellum, potentially sustaining the observed motor and cognitive impairments [34]. Within the cerebellum, GABAergic neurons are an essential modulator of signaling among GCs, mossy fibers, and PCs [37,50]. In the Ts65Dn mouse model, the GCs are more excitable and have a smaller tonic receptor current, leading to a proposed treatment of enhancing the tonic inhibition of GCs to improve coordination defects in DS [2,19].

Additionally, there was a notable decline in the number of cells within the cerebellum and the distance that cells migrated from the sphere relative to the euploid control. This decline was noted in numerous neuronal regions, including the subgranular zone of the dentate gyrus, the subventricular zone of the lateral ventricle, and the cerebellum, in both prenatal and postnatal life stages of the Ts65Dn mouse [77]. Though increased inhibitory GABAergic transmission in Ts65Dn cerebellums has been noted, an understanding of why is unclear; however, altered migration during early development has been proposed to be a possible cause [12,35]. During postnatal development of the WT cerebellar GCs, a decrease in size and frequency of the postsynaptic GABAergic currents that were spontaneously occurring while the tonic GABAergic current appears at ~ P7 and increases in magnitude so that in mature GCs, a majority of spontaneous GABAAR-mediated charge transfer occurs through the tonically active GABAARs [2]. In theory, the increase in tonic current would lead to overstimulation of the extrasynaptic 6GABAARs, leading to impaired motor coordination. It is unclear whether selecting 6GABAAR would help restore cerebellar function or would further the dysfunction. It is not yet understood if decreasing tonic current was a compensatory mechanism for reducing GCs in the cerebellum [2]. Early pharmacological research exemplifies the effect of 6GABAAR on motor action via the cerebellum, focusing on treating gait-specific neuropsychiatric disorders, thus strengthening the potential impact on patients with DS motor dysfunctions [76,78].

Table 1 summarizes the neuroanatomic and functional findings in Down syndrome.

**Table 1:** Summary of neuroanatomic and functional findings in Down syndrome

Category	Findings in Humans	Findings in Mouse Models	References
Imaging Findings	Reduced brain volumes (20% smaller); decreased frontal and temporal lobe volumes; reduced cerebellar size.	Decreased overall brain and cerebellar volumes (e.g., 88.1% reduction in Ts65Dn and Ts1Cje models).	[1,23,39,42,44]
Neuroanatomical Abnormalities	Reduced cortical folding, increased cortical thickness, and fewer neurons in layers II and IV.	Decreased granule cells (80% reduction in Ts65Dn); hypocellularity in granular and periventricular zones.	[7,17,23,51]
White Matter Integrity	Decreased myelination; reduced supratentorial white matter tracts.	Delayed oligodendrocyte differentiation; reduced white matter tracts; decreased nodes of Ranvier (early postnatal stages).	[1,18,44,48]
Motor Coordination Deficits	Delayed motor milestones; poor postural control; gait abnormalities.	Abnormal motor behaviours; increased hip and ankle stiffness; impaired postural control and gait dynamics in Ts65Dn and Ts1Cje models.	[36,59,62,63]
GABAergic Neurotransmission	Altered GABAergic inhibition; decreased GABA levels; reduced GABA receptor expression.	Delayed GABA developmental switch; increased inhibitory GABA transmission; impaired synaptic plasticity.	[5,19,34,37]
Therapeutic Implications	Treadmill training, core stability, and virtual reality improve motor coordination.	Pharmacological modulation of GABA alpha-5 receptors shows promise in restoring motor and cognitive deficits.	[15,49,64,65,70]

## Conclusions

Trisomy 21, a well-studied aneuploidy in neurology and developmental research, has been extensively linked to the characteristic symptoms of Down syndrome (DS). While the processes underlying these hallmark features are well-documented, motor and coordination deficits remain relatively underexplored. Despite widespread recognition of cerebellar atrophy and hypocellularity in DS patients and mouse models, the specific contributions of cerebellar dysfunction and GABAergic cell loss to these deficits warrant further investigation. This raises an important question: are motor coordination impairments merely associated symptoms or do they represent a core component of the diagnostic landscape? The GABAergic hypothesis offers a compelling framework to address this question, highlighting the role of cerebellar anomalies in motor dysfunction. Insights derived from DS mouse models and experimental pharmacological interventions provide a critical perspective, deepening our understanding of the cerebellar dysfunctions less frequently described in DS.
